# Long-term multidisciplinary integrative therapy management resulted in favorable outcomes for ovarian cancer during pregnancy: a case report and literature review

**DOI:** 10.1186/s13048-019-0584-3

**Published:** 2019-11-11

**Authors:** Tianmin Xu, Liang Wang, Yan Jia, Zanhui Jia, Zhuo Li, Shaohai Cui, Manhua Cui

**Affiliations:** Department of Gynecology and Obstetrics, the Second Hospital of Jilin University, Changchun, 130041 Jilin China

**Keywords:** Ovarian cancer, Pregnancy, Chemotherapy, Debulking surgery, Comprehensive treatment

## Abstract

**Background:**

Ovarian cancer during pregnancy is relatively rare and treatment strategies are inexperienced in surgery and chemotherapy. Multidisciplinary management of advanced epithelial ovarian cancer in pregnant patients with strong desire of fertility including sufficient mental and medical understanding, perioperative consideration, intraoperative decision, chemotherapy sensitivity and follow-up after treatment can gain successful outcomes for both maternal disease and fetus’s development.

**Case presentation:**

A 34-year-old primigravidae was diagnosed with advanced epithelial ovarian tumor and then first cytoreductive surgery to resect macroscopical lesions and protect the uterus for fetus was performed following with four chemotherapy courses (docetaxel and carboplatin) before delivery and four other chemotherapy courses after delivery. Chemotherapy drugs were decided by sensitivity test and the patient’s anaphylaxis. Second surgery involved cesarean section with a healthy offspring and secondary cytoreductive surgery. Operative strategies were considered to gain a balance of disease and risk for fetus. Psychosocial support was provided during the course of diagnosis and treatment for a healthy coping situation. This patient relapsed 19 months after the last chemotherapy course and was treated by additional adjuvant therapy to a clinical remission. The 33-month baby boy has no evidence with disease until now. The follow-up of both mother and baby is still continuing.

**Conclusions:**

Ovarian cancer during pregnancy has low incidence which must increase in future as women delay reproduction age. Ovarian cancer cytoreductive surgery and chemotherapy have limitation to handle conditions under a desire of fetus protection. Multidisciplinary treatment model is a therapeutic solution and a challenge for gynecological surgeons, medical oncologists, pathologists, obstetricians, neonatologists, pharmacists, anesthetist, and psychologists.

## Background

The incidence of pregnancy-associated cancer ranges from 0.02 to 0.1% [1]. The prevalence of adnexal masses in pregnancy is reported as 0.15–5.7% and most of them are benign, while reported incidence of ovarian cancer in pregnancy varies from 1 in 15,000 to 1 in 32,000 [2]. Statistically, ovarian cancer is the fifth most common cancer diagnosed during pregnancy, following breast, thyroid and cervical cancer and Hodgkin lymphoma [3]. For histological types, most of these tumors are germ cell tumors and epithelial ovarian malignancy is reported to occur in 1:12000 to 1:50000 pregnancies generally [4]. Due to low incidence of ovarian cancer during pregnancy, standard management has not been set up and teratogenic effects whether associated with chemotherapy drugs cannot be concluded totally. Because of delay of reproduction age of women, the incidence of malignancy during pregnancy is going to increase in future and fertility preservation is becoming a more major issue [5]. The goal for pregnant patients with malignancy is the same with non-pregnancy to improve free survival. But beyond that, an optimum balance between timing/approach of managing the mother’s cancer and preservation of the fetus is imperative. Treatment principles can refer to non-pregnancy and have unique characters depending upon stage, type, gestational age and metastatic spread. Overall survival and recurrence-free survival rates of malignancy patients during pregnancy are not worsened comparing with non-pregnant patients [6].

Overall, multidisciplinary care from obstetricians, gynecologists, oncologists, chemotherapists, pharmacists, pathologists, pediatricians, anesthetist and psychologists can give ovarian cancer patients comprehensive and long-term treatment during pregnancy. Herein, a case of an advanced epithelial ovarian cancer diagnosed at the second trimester with serous lesions after a complex but comparatively successful treatment is reported. Chemotherapy during pregnancy with docetaxel and carboplatin which was first administrated for ovarian cancer during pregnancy and a normal offspring was born in this case which showed experience of diagnosis, therapeutic timing, methods and negative effects of surgery and adjuvant therapy.

## Case presentation

**Fig. 1:**
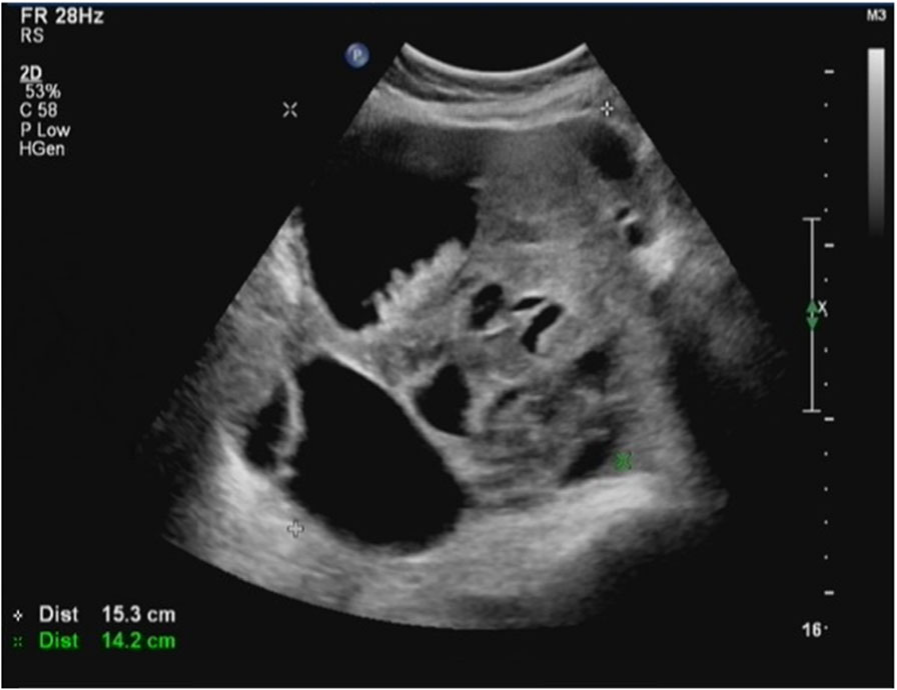
Ultrasound image at 20 weeks of gestation showed bulky masses behind uterus. The ultrasound image showed large cystic echoless mass behind uterus with obvious hyperechoic papillae in it

**Fig. 2:**
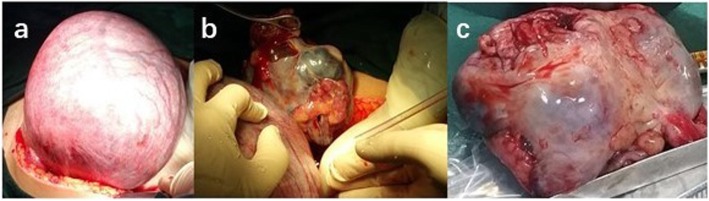
Macroscopic appearance of the uterus, the left ovary tumor during the first surgery. The middle trimester uterus was in normal size (**a**). The largest mass was on the left ovary behind the uterus whose surface was seriously crispy (**b**). Complete resection of tumor without rupture was performed (**c**)

**Fig. 3:**
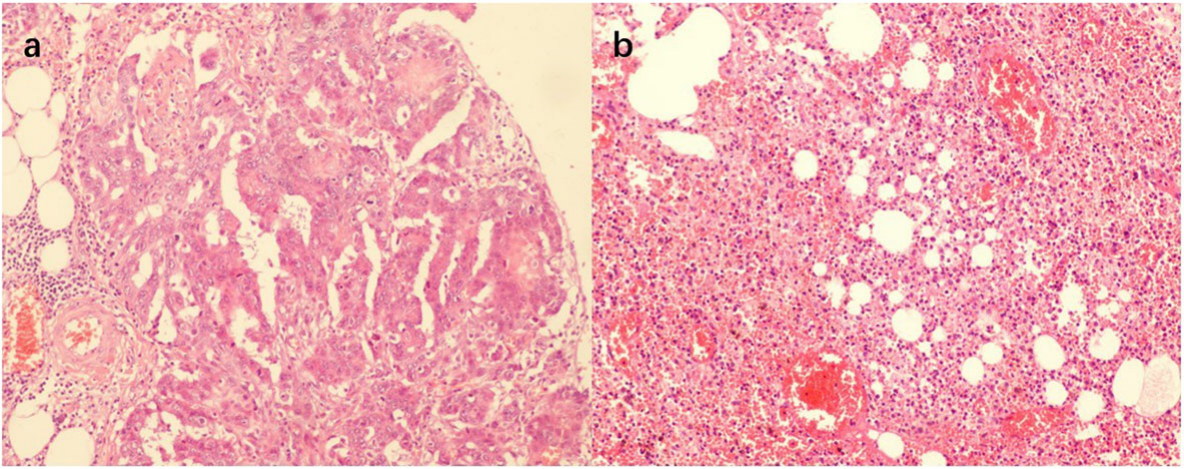
Microscopy pathological findings in the two surgeries. Typical high grade serous adenocarcinoma in left ovary in the first surgery (**a**). Hemorrhage, necrosis, inflammatory exudate, fibrinoid exudate in the lesions on the surface of bowel in the second surgery after four effective chemotherapy courses (**b**)

In January 2016, a 34-year-old primigravida presented on an ultrasound examination with cystic echoless mass (maximum diameter 15 cm) behind uterus and several hyperechoic papillae were detected in its inner surface (maximum diameter 5 cm) at 20 weeks of gestation (Fig. 1). Ultrasonography at 6 weeks and 18 weeks at gestation had found suspect pelvic mass in a local community hospital and she was enjoined regular examinations. The fetus was not with any malformation in ultrasound examination. The patient had no previous history of cancer in family. CA125, CA199, and HE4 levels were 125 U/ml, 31 U/ml, and 238 U/ml, respectively. Abdominal pain and obvious distention occurred. Careful examinations of other body systems were normal. Malignant tumor from ovarian cancer was highly suspicious and then exploratory laparotomy was performed at 21 weeks of gestation with combined spinal-epidural anesthesia (CSEA). Intraoperative condition was large amount of bloody abdominal ascites, extensive nodules (maximum diameter > 2 cm) involving the bowel, bladder, surface of uterus, omentum and peritoneum, bilateral ovaries had lesions (left maximum diameter 15 cm, right maximum diameter 8 cm) and left pelvis had been “frozen pelvic”. The uterus was in the normal size of the second trimester but the left ovary had a typical crispy surface (Fig. 2). Frozen section biopsy was positive for malignancy. This patient and her family had strong wish to preserve the fetus after they were told the potential risk for mother and fetus, the severe malignant degree of an advanced ovarian cancer and the subsequent changes of combinational therapy before operation by doctors in this multidisciplinary team. Conservation surgery was performed. Bilateral adnexectomy, infra-colic omentectomy, independent macroscopically visible resections of uterus surface lesions, peritoneal washing was performed. The patient and fetus had no complication during perioperative period. The final histopathological diagnosis was a high grade serous adenocarcinoma and the patient was staged at IIIC (FIGO) (Fig. 3a). This patient had a chemotherapeutic drug sensitivity test in vitro by cell biological approaches test which showed that the cancer cells are sensitive to Taxanes and Carboplatin.

At 22 weeks of gestation, the first course chemotherapy was administrated. At Taxol (paclitaxel) test volume, the patient self-described dyspnea and electrocardiogram showed sinus tachycardia. At last, Taxotere (docetaxel) and Paraplatin (cisplatin) was used in four courses of chemotherapy before delivery. No fetal anomalies were documented at several periodic ultrasounds during the four chemotherapy courses. After the second chemotherapy course, serum biomarker had dropped to normal range. The Chinese version of Cognitive Emotion Regulation Questionnaire (CERQ- C) was filled out by this patient and her partner in psychological aspect which showed a favorable inclination towards adaptive strategies and duration psychological support was provided.

At 35 weeks of gestation, cesarean section was performed with CSEA followed by radical surgery for ovarian cancer with general anesthesia. The male infant was 2100 g with Apgar scores of 8 and 9 at 1 and 5 min, respectively. The placenta appeared normal at the time of delivery. At surgery, residual lesions involved the uterine surface, Douglas’ pouch, sigmoid colon (maximum diameter 5 cm). Cytoreductive surgery for ovarian cancer was operated. Hysterectomy, pelvic/para-aortic lymphadenectomy, omentectomy, appendectomy, partial sigmoidectomy, peritoneal washing was performed and all apparent independent cancer nodules were resected. Final histopathological revealed negative lymphonodi and surgical margins of sigmoid colon (Fig. 3b). After delivery, the patient had four more chemotherapy courses. At the end of the last chemotherapy course, clinical examinations were without any evidence of disease.

This patient had recurrence 19 months after the last chemotherapy with PETCT examination showing several swollen pelvic lymph nodes and right supraclavicular lymph node and serum CA125, HE4 was 177.10 U/mL and 142 pmol/L, respectively. One cycle of TC regimen was administrated, but CA125 increased to 268.10 U/mL so DC regimen (PLD, pegylated liposomal doxorubicin and carboplatin) replaced. This patient had serious hypersensitivity reaction (hot flashes, dyspnea, tachycardia and facial numbness) during carboplatin intravenous infusion so DC regimen changed into PLD plus bevacizumab regimen. Until now, the patient has finished six courses of that regimen and there is no evidence of disease both in serum biomarkers and imageological examinations. The baby boy now 33-month old, is in fairly good condition and growing normally.

The patient provided informed consent to publish the case details and associated images.

## Discussions

The diagnosis of ovarian cancer mainly depends on ultrasonography routinely antenatal examinations during pregnancy. Pelvic magnetic resonance imaging (MRI) with gadolinium injection can be performed after the first trimester and should only be recommended when ultrasound examination doesn’t provide sufficient evidence for diagnosis [7]. Computed tomography (CT) is relatively contraindicated during pregnancy because of ionizing radiation’s potential teratogenic effect and concerns from patients and relatives. In this case, ultrasound had revealed pelvic masses at 6 weeks and 18 weeks of gestation but temporary observations had been done in view of treatment discretion for pregnancy and the patients’ wishes in the local hospital. Ultrasound at 20 weeks had showed large cystic echoless mass with obvious hyperechoic papillae highly suspecting malignancy. Most women with ovarian cancers during pregnancy are asymptomatic and some patients with unspecific symptoms such as vague abdominal pain, and/or abdominal distension which are common in normal pregnant women [8]. This patient complained of abdominal distension at the beginning of her medical treatment and was confirmed by surgery owing to a large amount of ascites. Blood markers to monitor ovarian cancers can be physiologically raised during pregnancy [1]. Abnormal level of CA125 is widely used in epithelial ovarian cancer detection and monitoring [9]. CA 125 is often elevated at 1st trimester and its level is low in maternal serum but high in the amniotic fluid in 2nd and 3rd trimester so it still has diagnosis value for 2nd and 3rd trimester [3]. In this case, the combined markers (CA125, CA199 and HE4) are obviously increased in the 2nd trimester which confirmed the significance of combined tumor markers detection and extent of increase of biomarkers for diagnosis. Diagnosis of ovarian cancer during pregnancy has more heedful identification than non-pregnancy according to clinical symptoms, imageological examination and serologic testing.

Evidence from published literature indicates that pregnancy does not significantly affect the prognosis of ovarian cancer [8]. The treatment goal of ovarian cancer during pregnancy is to achieve the best oncologic outcome and preserving the fetus viability. This patient was diagnosed in the 2nd trimester with a less risk of abortion yet in the 1st trimester functional corpus luteum is necessary for fetus. The aim of pregnancy sparing debulking surgery is to resect as many tumor lesions as possible to relieve symptoms meanwhile protecting the uterus to prevent fetal hypoxia. In order to prevent excessively pulling the uterus and also because of severe lesion invasion, excision of the bowels and resection of the pelvic cavity lesions (especially in the Pouch of Douglas) were not done. During the first surgery, palpation of pelvic lymph nodes was normal. The 2nd trimester is a vulnerable period for fetal brain development when exposed to maternal anesthesia [10] and CSEA is decided in the first surgery depending on extent of disease. However, there has not conclusive evidence from the outcome studies discussed above that the type of anesthetic technique (regional or general) influences pregnancy outcome and more studies about anesthetic are needed [11].

In this case, the second surgery included cesarean section and debulking surgery after four courses of chemotherapy before delivery. Three cases of ovarian cancer concerning placental metastasis has been reported but no case involved transmission to foetus probably because of trophoblasts-containing placental barrier, immune system of the foetus and ovarian cancer metastasizing predominantly via peritoneal dissemination not via blood vessels [12]. Vertical transmission from the mother’s malignancy during pregnancy to the fetus/baby some even years after delivery has been discovered in other malignancies and whether chemotherapy drugs can avoid metastasis to the products of conception does not have any conclusion [13]. For gynecologists, careful check of placenta is important during cesarean section to evaluate intraoperative metastasis and observe the development for the baby. Complete debulking surgery remained the basis of management of advanced ovarian cancer but when it followed cesarean section, serious bleeding and unclear operative field was a challenge for a grade IV surgery in this case. During debulking surgery, the disease had no progress in general, and the tumor lesions on intestine surface and pelvic cavity had been reduced obviously and the appearance of massive necrosis not tumor nodules of the intestinal surface lesions proved sensitivity to four chemotherapy courses.

Chemotherapy in the second and third trimester is associated to less risk of congenital malformation, intrauterine growth restriction or abortion than in the first trimester but some studies also report results from low number of patients that chemotherapy during the first trimester does not have obvious teratogenesis for fetus [14]. Chemotherapy drugs should be decided according to sensitivity test if possible. Taxanes plus carboplatin as the standard chemotherapy treatment for epithelial ovarian cancer under guideline [15]. Taxanes (paclitaxel and docetaxel) are mitotic poisons to disrupt the cellular microtubular network. According the patient’s symptoms at paclitaxel test volume, adverse infusion-related reactions happened due to Cremaphor EL which is an excipient for paclitaxel, and to switch from paclitaxel to docetaxel can avoid adverse reactions caused by excipients because paclitaxel and docetaxel are under the same antitumor mechanism, yet docetaxel diluent is polysorb 80 [16, 17]. Exposure to Taxanes during pregnancy has no direct or indirect evidence to harmful effects of fetus at delivery and at postnatal follow-up [18]. However, long-term follow-up study is necessary for the child physically and neurologically without limitation of years. For example, a case of attention deficit disorder in an 11-year old child exposed to paclitaxel in utero was reported [19]. An ex vivo model confirmed that carboplatin with regular doses is not associated with significant placental transfer, fetal exposure, or fetal toxic effects and cases receiving carboplatin during the second trimester had no serious effects on the fetus [20, 21]. This has been the only case using docetaxel combined with carboplatin for ovarian cancer during pregnancy until now (another case reported for lung cancer only one course before delivery), while paclitaxel combined carboplatin or single carboplatin are reported before [22, 23]. A 3 weeks interval is recommended between the last cycle of chemotherapy and the delivery because emission of hematopoietic suppression caused by chemotherapy drugs is expected for both mother and fetus [24]. In this case, cesarean section and radical surgery was carried out 25 days after the forth chemotherapy course to protect the perioperative blood parameters of mother and the stable condition of fetus.

The survival rates of stages III have survival rate ranges from 30 to 50% and the progression-free survival (PFS) of stage IIIC is 12 months in non-pregnant patients [25] [26]. This patient had a 19-month disease-free survival and was fully platinum sensitive (platinum-free interval at ≥12 months after initial chemotherapy) which was above the mean PFS [27]. The patient was not sensitive to Taxanes so substituted PLD was administrated. PLD has been extensively used in ovarian cancers and is as effective as non-liposomal Dox with a better cardiac safety profile [28]. There are results showed carboplatin-PLD regimen has better tolerance and more favorable safety than paclitaxel-carboplatin regimen [29]. Carboplatin hypersensitivity occurrence is related to more carboplatin treatment cycles and higher carboplatin doses and a higher incidence is among patients with advanced disease (stage III–IV) with serous carcinoma [30]. Addition of bevacizumab offers meaningful improvement in PFS and ORR (overall response rate) in ovarian cancer but it is not recommended with a lack of data regarding pregnant women [31]. Totally, chemotherapy for this patient during pregnancy and after delivery was generally successful considering pharmaceutical effect, drug metabolism and transportation, chemical teratogenesis and drug allergy and resistance by gynecologist, oncologists, pharmacists and embryologist.

In this case, the strong will to preserve the baby and burden the risk of malformation with treatment during pregnancy can be understand in culture and economy in a developing country. Married infertile women has lower overall and comprehensive quality of life scores and higher anxiety scores when compared with fertile controls in China [32]. Considering the particular stressful life event for this family, psychological support accompanied the long-term therapy with satisfied coping strategies. CERQ- C consists of 9 subscales to characterize the individual’s style of responding to negative events and CERQ-C showed internalizing adaptive cognitive emotion strategies in the patients and her partner which guided the attitude towards malignancy and fertility also the communication and trust between doctors and patients which is the foundation of clinical medicine [33, 34]. As for the baby’s development, psychological strategies can set the ground for construction of a relatively perfect mother-child relationship especially when the mother with malignancy [35].

In summary, we demonstrated successful outcomes for baby and mother produced after elaborate surgery and chemotherapy during pregnancy under the guidelines and clinical practical experience for individual considered by multidisciplinary doctors. This case was the first case reporting combination of docetaxel and carboplatin for ovarian cancer during pregnancy. Although the dilemma of preserving fetus and treating mother makes ovarian cancer during pregnancy different from non-pregnancy, treatment principle of maximal debulking surgery and chemotherapy for sensitive patients are almost similar. Protection of uterus in surgery, teratogenic effect of chemotherapy drugs, and psychological support and long-term follow-up are unique for ovarian cancer during pregnancy. As women delay childbearing to older ages, malignancy during pregnancy will gradually increase which will be a major challenge for multidisciplinary therapy and also a social issue involving economy and culture. Standard therapeutic strategies need to be set up with more cases as well as completed follow-up.

## Data Availability

The authors declare that the data supporting the findings of this study are available within the article.

## Abbreviations

CA125: Cancer antigen 125
CA199: Cancer antigen 199
CSEA: Combined Spinal-epidural Anesthesia
CT: Computed tomography
DC: Regimen Pegylated Liposomal Doxorubicin and Carboplatin Regimen
FIGO: International Federation of Gynecology and Obstetrics
HE4: Human Epididymal Protein
MRI: Magnetic Resonance Imaging
PFS: Progression-free Survival
PLD: Pegylated Liposomal Doxorubicin
